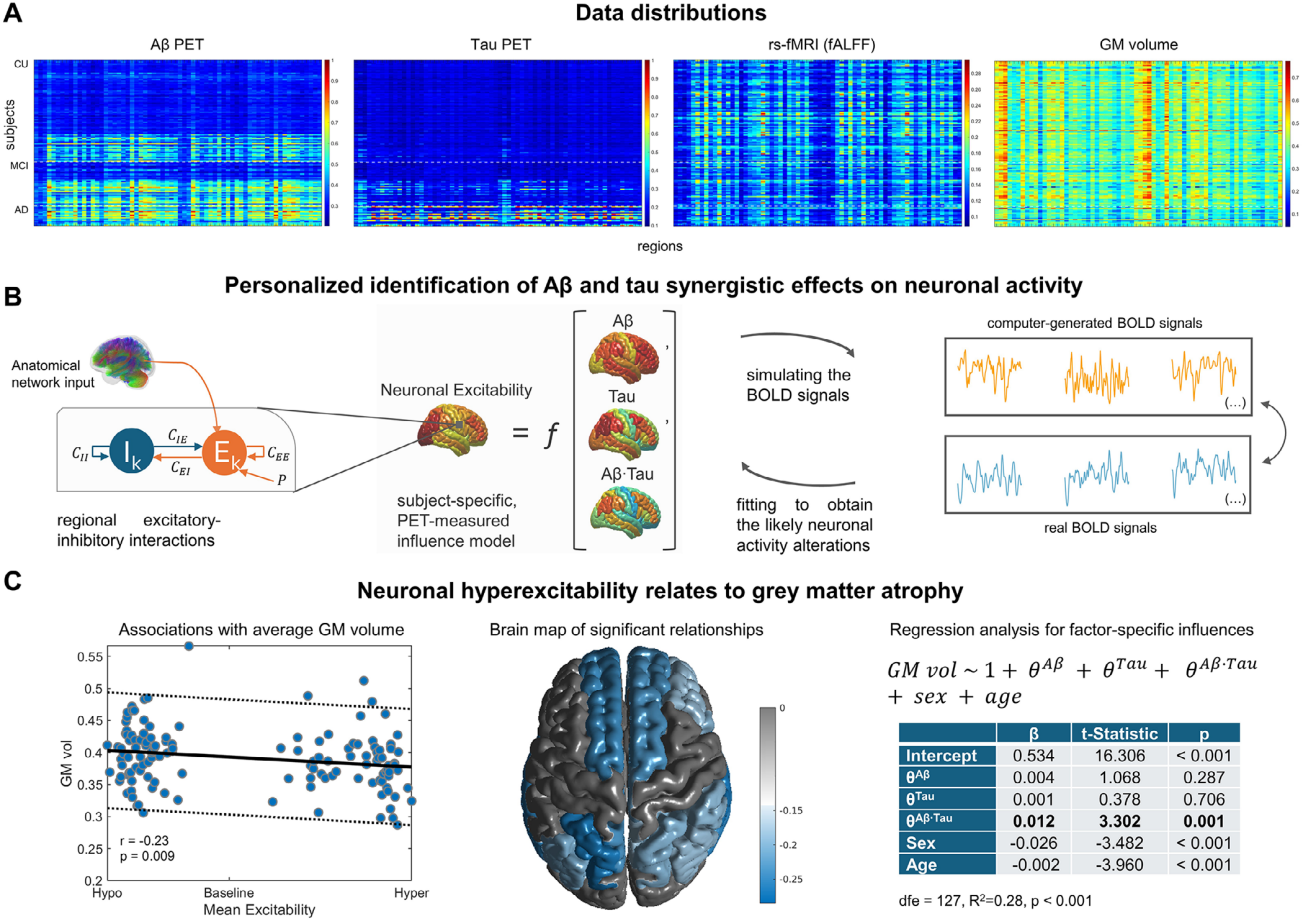# Aβ‐ and Tau‐Mediated Neuronal Excitability Derived from fMRI Predicts Grey Matter Atrophy in Alzheimer’s Disease

**DOI:** 10.1002/alz.090349

**Published:** 2025-01-03

**Authors:** Lazaro M Sanchez‐Rodriguez, Gleb Bezgin, Felix Carbonell, Joseph Therriault, Jaime Fernandez Arias, Stijn Servaes, Nesrine Rahmouni, Cécile Tissot, Jenna Stevenson, Pedro Rosa‐Neto, Yasser Iturria Medina

**Affiliations:** ^1^ McGill University, Montreal, QC Canada; ^2^ Biospective, Inc., Montreal, QC Canada

## Abstract

**Background:**

Despite amyloid‐β (Aβ) plaques and tau neurofibrillary tangles being recognized as major Alzheimer’s Disease (AD) hallmarks, their synergistic contribution to neuronal activity remains unclear. We developed a neuroimaging‐based personalized brain activity model to assess the *in‐vivo* functional impact of AD pathophysiology. In previous reports, model‐inferred neuronal excitability predicted disease progression (i.e., cognitive performance and certain biological markers) [1,2]. Here, we investigate its relationship with brain tissue atrophy.

**Method:**

We used Aβ and tau PET scans, structural T1‐weighted 3D MRI and resting‐state functional MRI from 132 subjects in the TRIAD cohort (https://triad.tnl‐mcgill.com/, Fig. 1a). The participants were diagnosed as cognitively unimpaired (CU, N = 81, 71.15 ±7.73 yrs, 63 F), mild cognitive impairment (MCI, N = 35, 72.1±7.84 yrs, 17 F) or Alzheimer’s disease (AD, N = 16, 69.45 ± 9.10 yrs, 8 F).

In our whole‐brain neuronal activity model, the individual regional Aβ and tau burdens mediate neuronal excitability (Fig. 1b). By maximizing the similarity between simulated and real resting‐state signals, we estimated the contributions by Aβ, tau and their interaction (Aβ∙tau). A post‐hoc correlation analysis sought to determine if the reconstructed excitability values aligned with grey matter atrophy assessed via voxel‐based morphometry.

**Result:**

Fig. 1c (left) presents the relationship between average intra‐brain grey matter volume and the obtained excitability values. Reduced grey matter volume significantly associated with the participants’ neuronal hyperactivation. Such a behaviour was additionally observed in region‐specific analyses, particularly at brain regions with grey matter alterations by AD, including the parahippocampal gyrus, fusiform gyrus, amygdala, hippocampus, entorhinal cortex and posterior cingulate gyrus (Fig. 1c, center). The effect of Aβ and tau’s interaction on neuronal excitability was the only variable of interest predicting grey matter volume (Fig. 1c, right).

**Conclusion:**

The computationally derived excitability values inversely correlated with grey matter volumes at the brain regions that most prominently showcase neurodegeneration in AD. Our results are consistent with previous observations [1,2] of increased excitability in parallel with pathological spread and the loss of neurons in the AD brain, also highlighting the fundamental role played by Aβ and tau functional interactions.

**References**

[1] Sanchez‐Rodriguez et al, 2023 https://www.ncbi.nlm.nih.gov/pmc/articles/PMC10370127

[2] Sanchez‐Rodriguez et al, 2023 https://doi.org/10.1101/2023.09.15.557737